# Fractal dimension analysis of resting state functional networks in schizophrenia from EEG signals

**DOI:** 10.3389/fnhum.2023.1236832

**Published:** 2023-09-20

**Authors:** Juan Ruiz de Miras, Antonio J. Ibáñez-Molina, María F. Soriano, Sergio Iglesias-Parro

**Affiliations:** ^1^Software Engineering Department, Research Center for Information and Communication Technologies (CITIC-UGR), University of Granada, Granada, Spain; ^2^Department of Psychology, University of Jaén, Jaén, Spain; ^3^St. Agustín University Hospital, Linares, Jaen, Spain

**Keywords:** fractal dimension, EEG, resting state, functional network, schizophrenia

## Abstract

Fractal dimension (FD) has been revealed as a very useful tool in analyzing the changes in brain dynamics present in many neurological disorders. The fractal dimension index (FDI) is a measure of the spatiotemporal complexity of brain activations extracted from EEG signals induced by transcranial magnetic stimulation. In this study, we assess whether the FDI methodology can be also useful for analyzing resting state EEG signals, by characterizing the brain dynamic changes in different functional networks affected by schizophrenia, a mental disorder associated with dysfunction in the information flow dynamics in the spontaneous brain networks. We analyzed 31 resting-state EEG records of 150 s belonging to 20 healthy subjects (HC group) and 11 schizophrenia patients (SCZ group). Brain activations at each time sample were established by a thresholding process applied on the 15,002 sources modeled from the EEG signal. FDI was then computed individually in each resting-state functional network, averaging all the FDI values obtained using a sliding window of 1 s in the epoch. Compared to the HC group, significant lower values of FDI were obtained in the SCZ group for the auditory network (*p* < 0.05), the dorsal attention network (*p* < 0.05), and the salience network (*p* < 0.05). We found strong negative correlations (*p* < 0.01) between psychopathological scores and FDI in all resting-state networks analyzed, except the visual network. A receiver operating characteristic curve analysis also revealed that the FDI of the salience network performed very well as a potential feature for classifiers of schizophrenia, obtaining an area under curve value of 0.83. These results suggest that FDI is a promising method for assessing the complexity of the brain dynamics in different regions of interest, and from long resting-state EEG signals. Regarding the specific changes associated with schizophrenia in the dynamics of the spontaneous brain networks, FDI distinguished between patients and healthy subjects, and correlated to clinical variables.

## 1. Introduction

To respond to the demands of a constantly changing environment the brain permanently adjusts its architecture, alternating between segregated functioning, as when performing highly automated tasks, and integrated functioning, as when performing tasks that require high cognitive effort (Shine and Poldrack, 2018). This neural adaptation spans multiple time scales, and different regions. In the present work, we employ a metric (FDI), developed in our laboratory (Ruiz de Miras et al., 2019), that captures the dynamics of the complexity of brain activity to try to learn more about the severe mental disorder of schizophrenia.

The fractal dimension (FD; Mandelbrot, 1983) is a quantitative measure of shape complexity used widely for analyzing electroencephalogram (EEG) signals in many neuropsychiatric disorders (Lau et al., 2022). The fractal dimension index (FDI; Ruiz de Miras et al., 2019) is a recent methodology designed for analyzing the complexity of the spatiotemporal dynamics in the brain. FDI is based on computing the 3D fractal dimension (3DFD) and the 4D fractal dimension (4DFD) of cortical activations extracted from the EEG signal. The 3DFD and 4DFD values are computed from the 3D point clouds defined by the cortical activations, induced by a transcranial magnetic stimulation, at each time sample of the epoch being analyzed. FDI is calculated as the product of two different components: the Higuchi fractal dimension (Higuchi, 1988) of the evolution of the 3DFD values in the epoch, noted as HFD (3DFD), and the 4DFD of the 4D representation obtained by putting together all of the 3D point clouds contained in the whole epoch (time is the fourth dimension). The 4DFD component in FDI quantifies the integration of cortical networks, while the HFD (3DFD) component is a measure of the cortical differentiation (Ruiz de Miras et al., 2019).

Schizophrenia is a severe mental disorder whose patients suffer psychotic symptoms and several cognitive and motor dysfunctions (McGrath et al., 2008). The symptoms’ onset is usually at between 14 and 30 years of age, and it is known that the time between the manifestation of symptoms and treatment is one of the best predictors of later prognosis (McGlashan, 1999). Currently, there are no reliable biomarkers for schizophrenia and, therefore, providing tools for an early diagnosis is of great relevance (Nieuwenhuis et al., 2012).

In the last few years, several studies used complexity measures of EEG for analyzing neural activity in schizophrenia (Harmah et al., 2020; Lau et al., 2022). Change complexity was used in Aksentijevic et al. (2021) for analyzing both the spatial and temporal complexity of 16 EEG channels. Kutepov et al. (2020) compared EEG complexity between schizophrenia patients and healthy controls by using three measures of entropy (approximated entropy, sample entropy, and multi-scale entropy). Another entropy measure of EEG, fuzzy entropy, was used in Xiang et al. (2019) for measuring task-related modulation of complexity in schizophrenia. Some other authors studied the complexity of EEG signals in schizophrenia by means of Higuchi’s fractal dimension (HFD; Portnova and Atanov, 2018; Goshvarpour and Goshvarpour, 2020). Fractal analysis of EEG signals was also used for extracting several features, such as HFD and correlation dimension, in recent schizophrenia classifiers based on different machine learning algorithms (Barros et al., 2021; Tian et al., 2022; Ruiz de Miras et al., 2023a). Multifractal and entropy-based features extracted from time series of topological measures (connectivity, clustering, and global efficiency) of functional networks constructed from EEG signals were also successfully used in classifying schizophrenia (Racz et al., 2020).

Increasing evidence suggests that schizophrenia is associated with alterations in the patterns of connectivity observed between different brain regions (Alexander-Bloch et al., 2010; Van Den Heuvel et al., 2010; Van Den Heuvel and Fornito, 2014; Iglesias-Parro et al., 2023). Understanding the complexity of the functional connectivity of neural networks in schizophrenia is crucial not only for understanding the mechanism of schizophrenia, but also for providing potential biomarkers for clinical use. Specifically, several authors have proposed that the pathophysiology of schizophrenia may be associated with changes of integration/segregation patterns in distributed neural networks, such as the default mode network (Wang et al., 2015) and the salience network (Huang et al., 2022). So complexity analyses of the neural activity in those known resting-state networks affected by schizophrenia could also be of great relevance.

In the present study, we hypothesized with the capacity of FDI to be adapted for analyzing both long recordings of resting-state EEG signals and localized regions of interest of the brain. To this aim, using the FDI measure, we analyzed the spatio-temporal complexity of brain dynamics for resting-state functional networks in schizophrenia. Our results demonstrated the ability of FDI to be applied on resting-state EEG signals from different regions of interest and revealed a localized decreasing complexity of neural activity of schizophrenia patients, mainly in the salience network.

## 2. Methods

### 2.1. Subjects

Our study included 31 subjects (see Table 1 for demographics and clinical data): 20 healthy control subjects (HC) and 11 individuals suffering from schizophrenia (SCZ). This dataset was recorded in previously-published studies of our laboratory, so the procedures for recruitment, acquisition and pre-processing of the data were described in prior publications (Iglesias-Parro et al., 2023; Ruiz de Miras et al., 2023a). In this section, those procedures are briefly described.

**Table 1:** Demographic and clinical data.

	SCZ	HC	Test, *p* value
*N*	11	20	
Age (years)	36.2 ± 10.2	40.7 ± 11.9	*U* = 85.0, *p* = 0.31^a^
Sex (M:F)	9:2	13:7	χ^2^ = 0.97, *p* = 0.32^b^
Education (Prim.:Second.:High)	2:8:1	1:12:7	χ^2^ = 3.29, *p* = 0.19^b^
SCIP-S—VLi	16.2 ± 3.9	21.1 ± 4.4	*U* = 32.5, *p* < 0.01^a^
SCIP-S—VLd	5.8 ± 2.1	6.8 ± 2.4	*U* = 75.0, *p* = 0.22^a^
SCIP-S—VF	13.6 ± 4.5	17.9 ± 4.1	*U* = 48.0, *p* < 0.05^a^
SCIP-S—WM	16.7 ± 4.0	19.5 ± 3.0	*U* = 60.5, *p* = 0.06^a^
SCIP-S—PS	7.6 ± 3.0	11.3 ± 2.9	*U* = 40.5, *p* < 0.01^a^
PANNS-P	13.9 ± 4.5		
PANNS-N	19.3 ± 8.2		
PANNS-G	29.4 ± 7.1		

Values expressed as mean ± standard deviation. SCIP-S, Screen for cognitive disability in psychiatry, Spanish version; VLi, Immediate verbal learning; VLd, Delayed verbal learning; VF, Verbal fluency; WM, Working memory; PS, Processing speed; PANSS, Positive and negative syndrome scale; PANSS-P, PANSS—positive subscale; PANSS-N, PANSS—negative subscale; PANSS-G, PANSS—general subscale. ^a^Mann–Whitney test; ^b^χ^2^ test.

Schizophrenia patients were recruited at the St. Agustín Hospital (Linares, Spain), and healthy subjects were recruited from students and staff of the University of Jaén (Jaén, Spain), the San Agustin Hospital (Linares, Spain) and from an Adult School (Jaén, Spain). The inclusion criteria for the SCZ group were a diagnosis of schizophrenia (F20), psychotic disorder (F23), or schizophrenic disorder (F25), according to the International Statistical Classification of Diseases, 10th revision. The exclusion criteria for both groups were diagnosis of mental disorder, substance abuse, history of developmental disability, vision disorders, or hearing disorders.

Cognitive functioning was measured using a Spanish adaptation of the Screen for Cognitive Disability in Psychiatry (SCIP-S; Pino et al., 2014) in both groups. SCIP-S provides a score for each of the following subscales: immediate and delayed verbal learning (VLi and VLd), verbal fluency (VF), working memory (WM), and processing speed (PS). In order to assess psychopathology in the SCZ group the Spanish version of the Positive and Negative Syndrome Scale (PANSS) was used (Kay et al., 1989; Peralta and Cuesta, 1994). The PANSS evaluates the schizophrenic syndrome with 30 items in three subscales: the positive subscale (seven items, PANSS-P), the negative subscale (seven items, PANSS-N), and the general psychopathology subscale (16 items, PANSS-G). Descriptive measures for all these measures are shown in Table 1.

### 2.2. EEG acquisition and pre-processing

Resting-state EEG data were recorded in a session where the subject was sitting in a comfortable chair looking at a cross placed in the center of the screen of a laptop. EEG was collected with a BrainAmps amplifier equipped with 32 channels following the standard 10–20 montage. EEG signals were recorded at a frequency of 500 Hz.

EEG pre-processing was performed in EEGLAB (Delorme and Makeig, 2004). As part of the signal preprocessing, we first applied a bandpass filter with cutoff frequencies of 1 and 30 Hz. Artifacts were extracted using infomax Independent Component Analysis (ICA) using runica.m MATLAB software. We use the default parameters of the runica.m algorithm, including the finalization of the learning process when weight-change <1e−06 or after 512 steps. A trained researcher removed segments with artifacts by visual inspection of the scalp topography, power spectra and raw activity from all ICA components, therefore, no automated artifact removal precedent was used. This process was carried out for each participant individually. For each participant, we finally selected 150 s of continuous clean data.

Source modeling was performed using the Brainstorm software (Tadel et al., 2011). First, the boundary element method implemented in OpenMEEG (Gramfort et al., 2010) was used to compute a forward EEG model. In this process the MNI/ICBM152 brain template of Brainstorm was used as the MRI anatomy (Fonov et al., 2009). Then, the inverse method sLORETA (Pascual-Marqui, 2002) as implemented in Brainstorm was used to obtain a source model of 15,002 current dipoles whose orientations were constrained from normal to cortex. The result of this source modeling process was a matrix of currents for each subject with a size of 15,002 (sources) × 75,000 (time samples).

### 2.3. Fractal dimension index computation

The fractal dimension index (FDI) was computed from the EEG data following a similar approach to that described in Ruiz de Miras et al. (2019). FDI was designed in Ruiz de Miras et al. (2019) for processing very short fragments of EEG data (130 milliseconds) where brain activations were induced from a transcranial magnetic stimulation. However, in the present study we had to adapt the processing of FDI to two key characteristics of resting-state EEG data: a much longer duration of the EEG records, and the absence of any stimulus which would help to identify brain activations.

**Figure 1:**
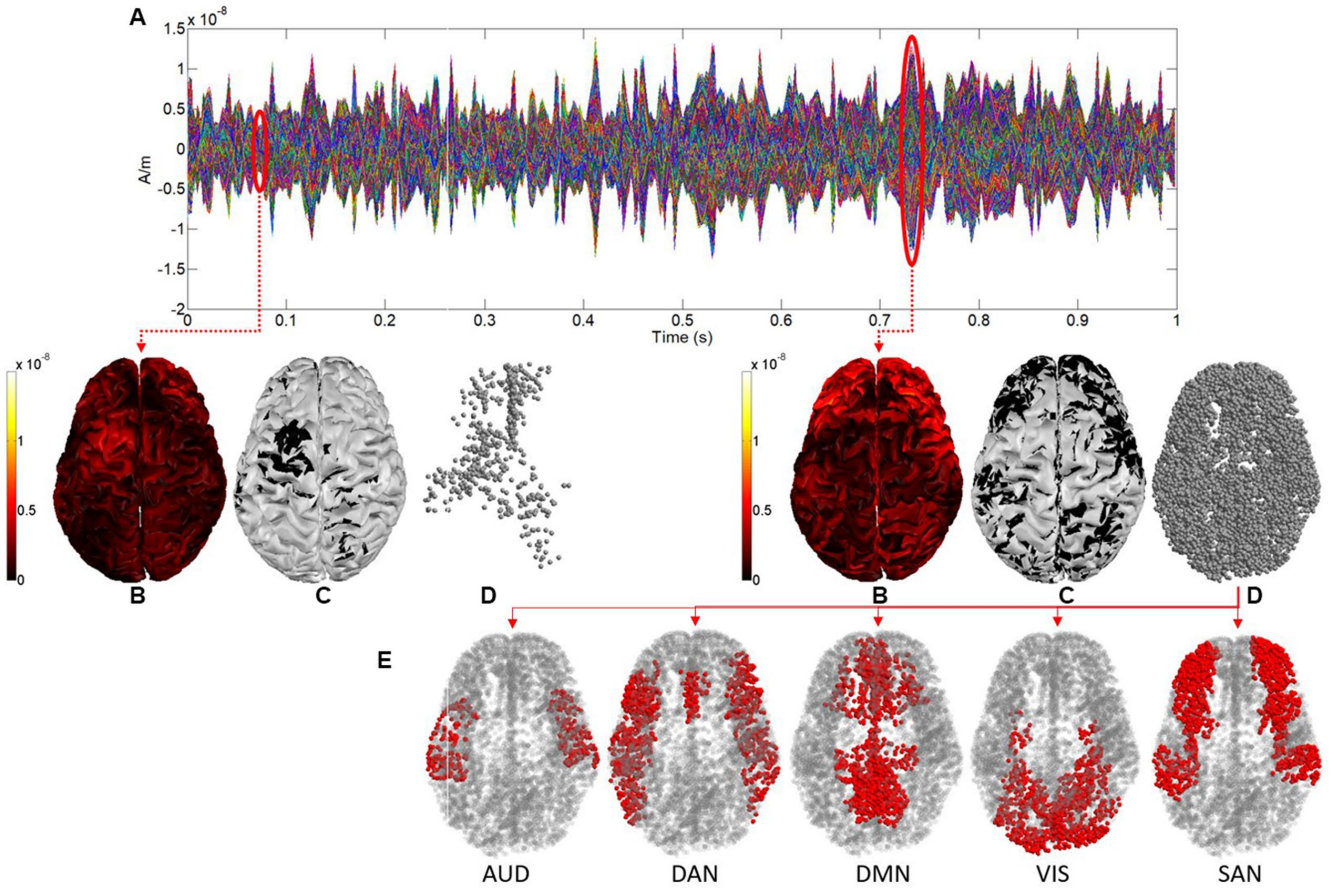
**(A)** Sources activity for 1 s. **(B)** Representation in 3D of the sources activity at two different time samples (0.06 and 0.73 s respectively). **(C)** 3D representation after binarization. **(D)** Point clouds defined by sources after binarization (cortical activations). **(E)** Cortical activations in each functional network (shown for the time sample of 0.73 s).

In order to deal with EEG records of 150 s, the whole epoch was divided using a sliding window of 1 s without overlapping, and then the FDI was computed for each 1-s window. Secondly, cortical activations at a time sample were defined as those sources (see Figures 1A,B) whose absolute values were greater than the mean plus the standard deviation of the absolute values of the corresponding current for all time samples in the epoch (see Figures 1C,D). By using this binarization threshold (mean + standard deviation) we selected as cortical activations at a time sample those sources whose value was significantly greater than the average in the epoch. Moreover, this threshold allowed us to select a sufficient amount of brain activations for each time sample, which guarantees a correct computation of the fractal dimension through the box-counting algorithm, both in 3D and 4D.

**Figure 2:**
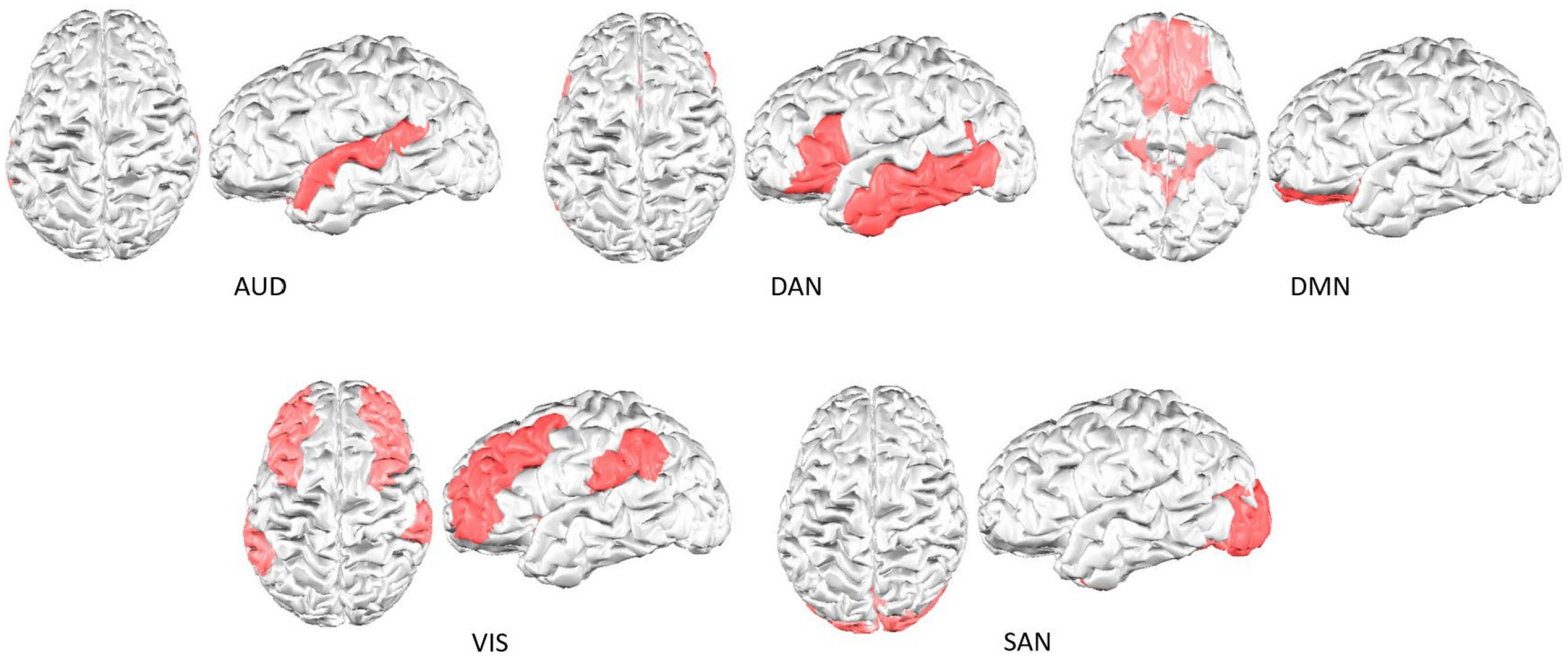
Cortical parcellation of the resting-state functional networks analyzed.

Finally, the brain activations in each resting state functional network were considered and processed separately, as shown in Figure 1 (cortical activations at time sample 0.73 s are colored in red for each network). Figure 2 shows the cortical parcellation that we used to delimit the functional networks. This parcellation, as proposed previously in Kabbara et al. (2017), defined five resting-state functional networks: the auditory network (AUD), the dorsal attention network (DAN), the default mode network (DMN), the salience network (SAN), and the visual network (VIS).

**Figure 3:**
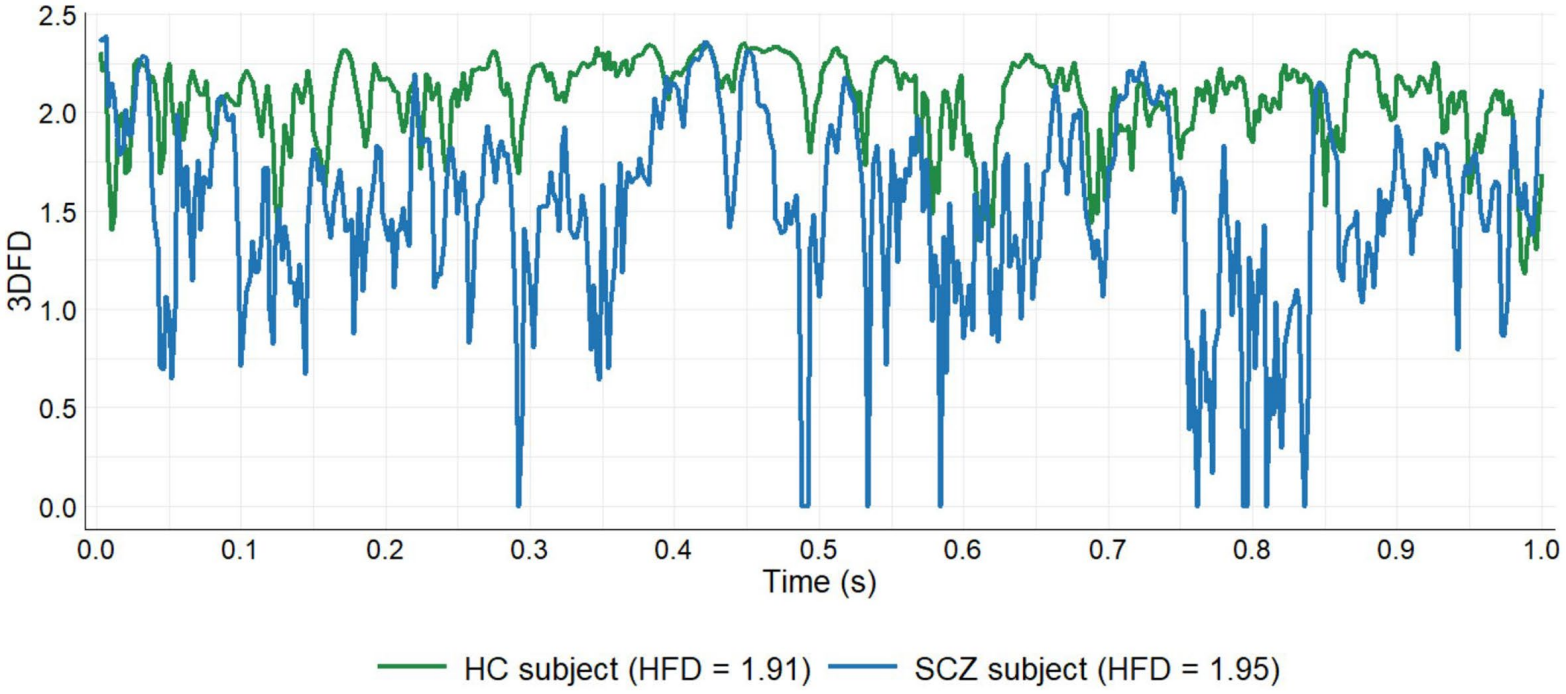
3DFD evolution for 1 s comparing a healthy subject and a patient. HFD, Higuchi fractal dimension of the curve described by the 3DFD values.

The FDI approach is based on two different fractal dimension algorithms: the box-counting algorithm (Russell et al., 1980) and the Higuchi fractal dimension (Higuchi, 1988). The box-counting algorithm is used to compute the fractal dimension of the point clouds defined by brain activations in both 3D and 4D. A 3D fractal dimension (3DFD) value was obtained from the 3D point cloud defined by brain activations at each sample of time (see Figure 1D). Moreover, the set of cortical activations (3D point clouds) in each 1-s window describes a 4D point cloud, where time is the fourth dimension. Again, the box-counting algorithm was used to compute the 4D fractal dimension (4DFD) value of this 4D point cloud. The 3DFD values in each 1-s window describe a curve as shown in Figure 3. Then Higuchi’s algorithm was used to compute the fractal dimension of this curve, noted HFD (3DFD). Therefore, for each 1-s window two different fractal dimension values were considered: 4DFD and HFD (3DFD). Finally, the FDI value of the window was obtained as the product of both quantities: FDI = 4DFD × HFD (3DFD). 4DFD values range from 3 to 4, and HFD (3DFD) values range from 1 to 2, so FDI values should range from 3 to 8. Full details of the FDI computation process can be found in Ruiz de Miras et al. (2019).

A total of 150 FDI values were obtained for each resting-state network of each subject. Each FDI consisted of 500 3DFD computations (one for each time sample) and a 4DFD computation. So we computed a total of 500 samples × 150 windows × 31 subjects × 5 networks = 11,625,000 3DFD values, and 150 windows × 31 subjects × 5 networks = 23,250 4DFD values. Since computing the fractal dimension in both 3D and 4D is a highly time-consuming task, we needed to use a parallel version of the box-counting algorithm optimized for its execution in the graphics processing unit (GPU) whose CUDA source code is provided in Ruiz de Miras et al. (2023b). On the other hand, the binarization process for obtaining the cortical activations from the Brainstorm matrices containing the source modeling were computed using custom MATLAB scripts.

### 2.4. Statistical analysis

The nonparametric Mann–Whitney *U* test (Mann and Whitney, 1947) and Chi-squared test (Pearson, 1900) were used to compare demographic and cognitive variables between groups. The non-parametric Quade’s analysis of covariance (ANCOVA; Quade, 1967), controlling for age, gender, and educational level, was used to study the differences in mean values of FDI between groups, with a Bonferroni post-hoc correction for multiple comparisons. Correlations between FDI and cognitive and psychopathology scores were assessed using the nonparametric Spearman correlation test (ρ; Spearman, 1904) with a Bonferroni *post hoc* correction for multiple comparisons. The potential use of FDI as a feature for classification algorithms was measured by using a receiver operating characteristic (ROC) curve analysis (Hanley and McNeil, 1982), where the proportion of true positive rate (sensitivity) was plotted against the proportion of false positive rate (1-specificity) at different levels of cumulative FDI values. The classification accuracy of FDI was assessed using the area under the ROC curve (AUC). The statistical analyses were performed in IBM SPSS 28, and the results were considered significant when the value of *p* of the statistical test was below 0.05.

## 3. Results

### 3.1. Demographic analysis

No significant differences were found between HC and SCZ groups in sex, age, and education (see Table 1). These results confirmed that both groups were matched regarding demographic variables. As can be also seen in Table 1, the HC group scored better than the SCZ group on all the SCIP-S cognitive tests, the difference being significant in all of the subscales except for delayed verbal learning and working memory.

### 3.2. FDI comparison between HC and SCZ

**Figure 4:**
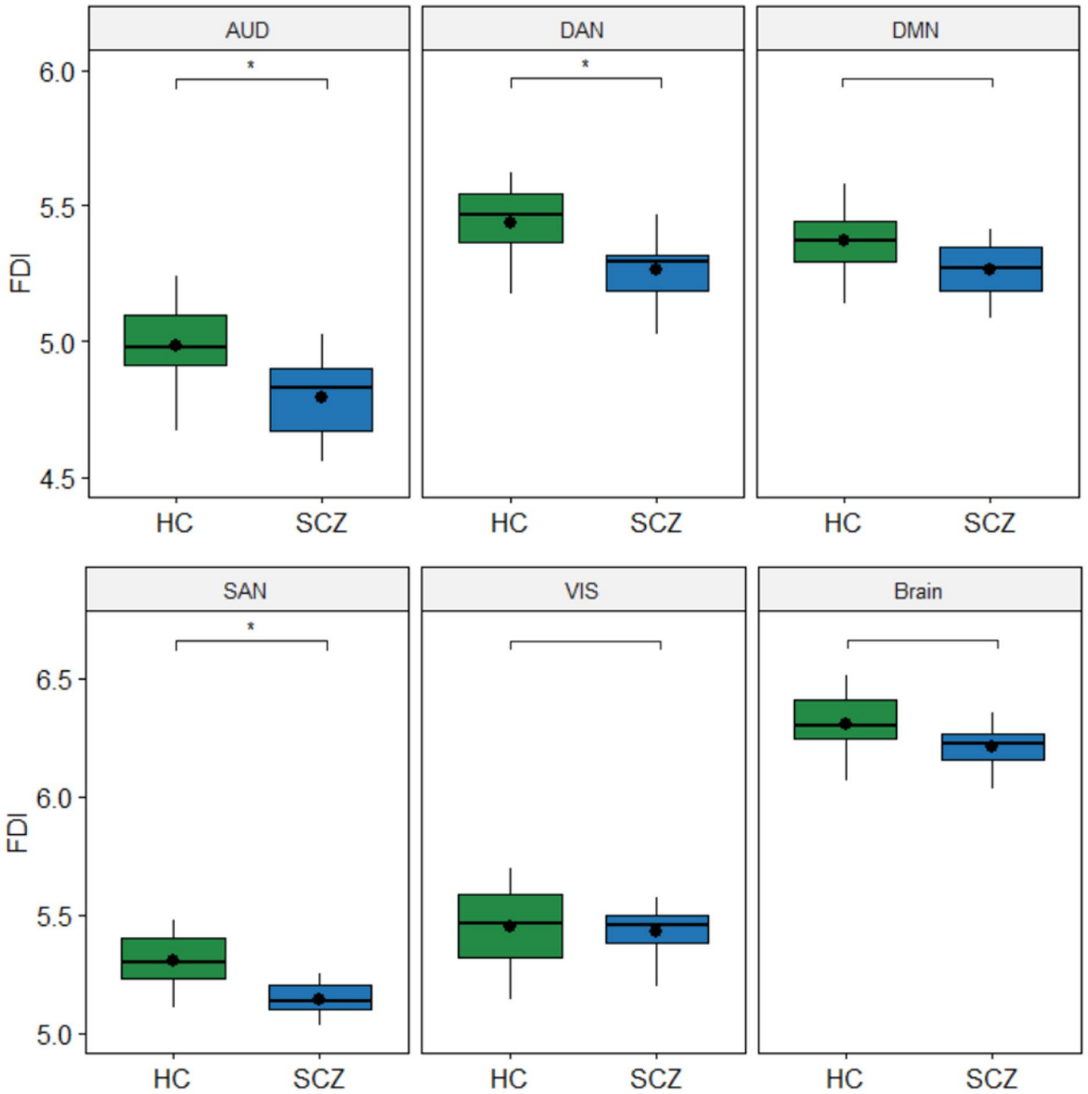
Boxplots with differences in fractal dimension index (FDI) between groups. The FDI values in each group are the average of the 150 FDI values computed for each subject in the corresponding network. Values of *p* for the Quade’s ANCOVA test controlling for age, gender, and education (^*^p < 0.05). Values of *p* were corrected for multiple comparisons with the Bonferroni *post hoc* method. No significant differences were found between groups for the whole Brain nor the DMN and VIS networks.

Figure 4 shows the boxplots comparing FDI between HC and SCZ groups for each resting-state network. For each subject in the corresponding network, the average of the 150 FDI values computed for all 1-s windows in the epoch was considered as a single FDI value. Results showed that FDI was significantly lower in the SCZ group for networks AUD (*F* = 7.72, *p* < 0.05), DAN (*F* = 7.33, *p* < 0.05), and SAN (*F* = 8.19, *p* < 0.05). No significant differences were found between groups for the whole brain (*F* = 3.48, *p* = 0.22) nor the networks DMN (*F* = 3.48, p = 0.22) and VIS (*F* = 0.19, *p* = 0.66).

**Figure 5:**
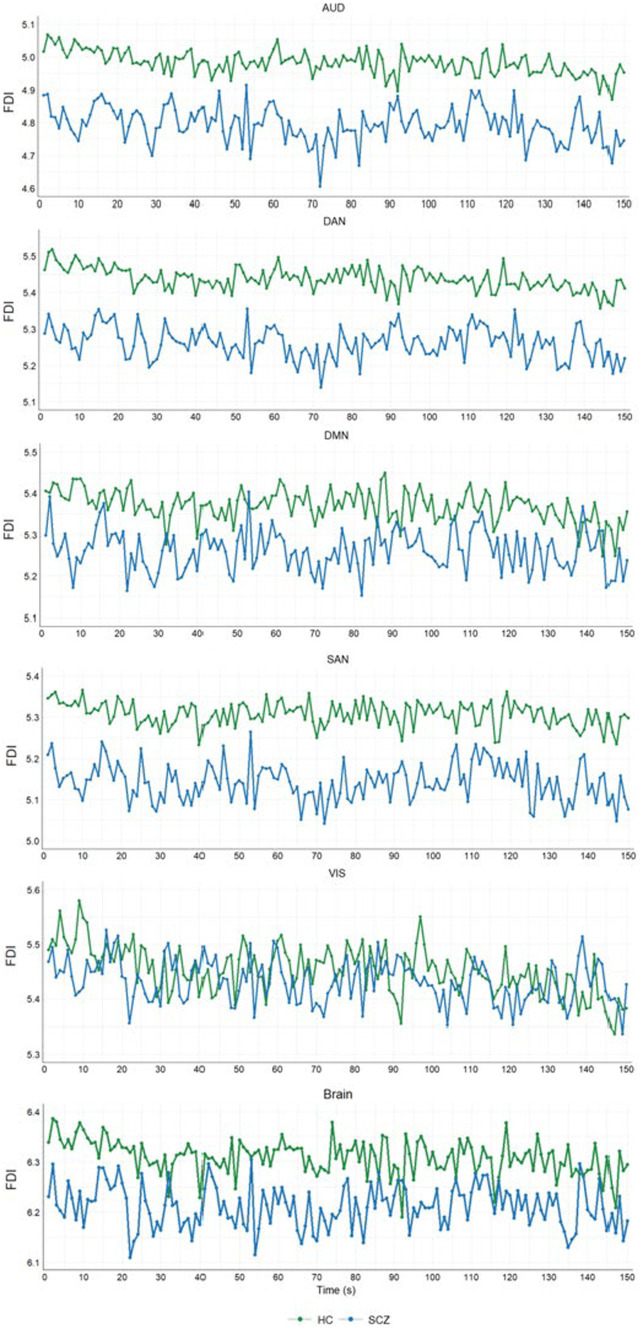
FDI evolution in 150 s for HC and SCZ groups. Each FDI point is the average of the FDI values of all the subjects of the group for the corresponding second.

We also analyzed the trends of the average FDI in each group for the 150 s of each epoch. Figure 5 shows these trends for each resting-state network and the whole brain comparing HC and SCZ groups. In this figure, each point corresponds to the average of the FDI values obtained in the corresponding 1-s window for all subjects of the group. Results showed that average FDI values were always lower in the SCZ group during the 150 s for all of the networks except VIS and the whole brain. For these two cases, the FDI trends of the HC and SCZ groups crossed each other at several points (see VIS and Brain panels in Figure 5).

### 3.3. Correlations between FDI and cognitive and psychopathological variables

**Figure 6:**
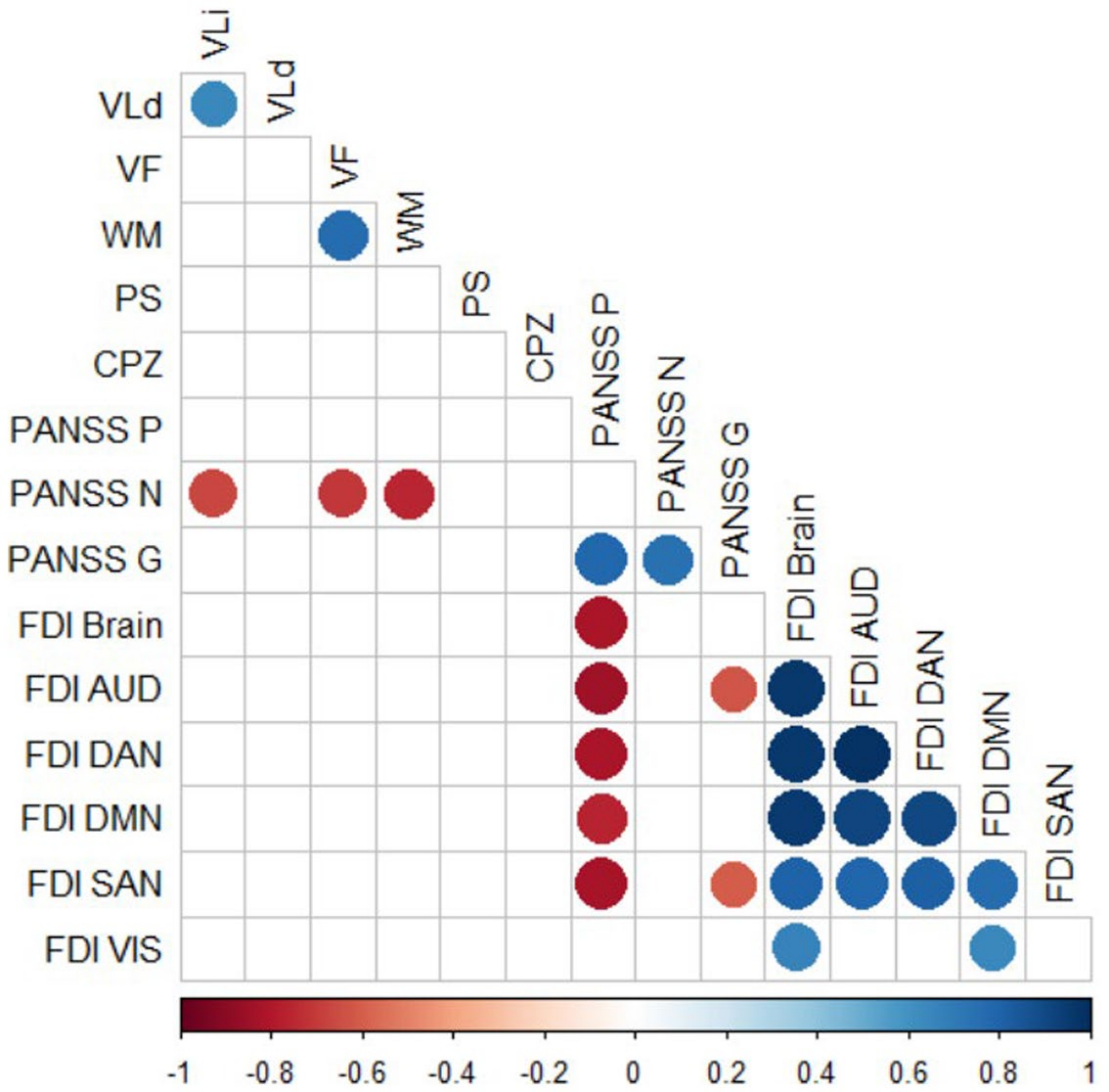
Significant Spearman correlations between cognitive and psychopathological scores and FDI in the SCZ group. Values of *p* were corrected for multiple comparisons with the Bonferroni *post hoc* method. Only significant correlations (*p* < 0.05) are shown. VLi, Immediate verbal learning; VLd, Delayed verbal learning; VF, Verbal fluency; WM, Working memory; PS: Processing speed; PANSS, Positive and negative syndrome scale; PANSS-P, PANSS—positive subscale; PANSS-N, PANSS—negative subscale; and PANSS-G, PANSS—general subscale.

Figure 6 shows the significant correlations between cognitive and psychopathological scores and the FDI obtained in each resting-state network and the whole brain for the subjects in the SCZ group. Details of significant Spearman correlations (ρ) and their corresponding values of *p* after Bonferroni correction for multiple comparisons are shown in Table 2. Significant strong negative correlations were found between PANSS-P and FDI in the whole brain and all resting-state networks, except VIS. Also, PANSS-G and FDI were significantly correlated in networks AUD and SAN.

**Table 2:** Details of significant correlations between psychopathological scores and FDI measures shown graphically in Figure 6.

FDI variable	Psychopathological variable	Correlation coefficient (ρ)	*p* value
FDI AUD	PANSS-P	−0.84	*p* < 0.01
FDI DAN		−0.81	*p* < 0.01
FDI DMN		−0.76	*p* < 0.01
FDI SAN		−0.82	*p* < 0.01
FDI Brain		−0.81	*p* < 0.01
FDI AUD	PANSS-G	−0.62	*p* < 0.05
FDI SAN		−0.61	*p* < 0.05

### 3.4. FDI as a potential feature for classifiers of SCZ

**Figure 7:**
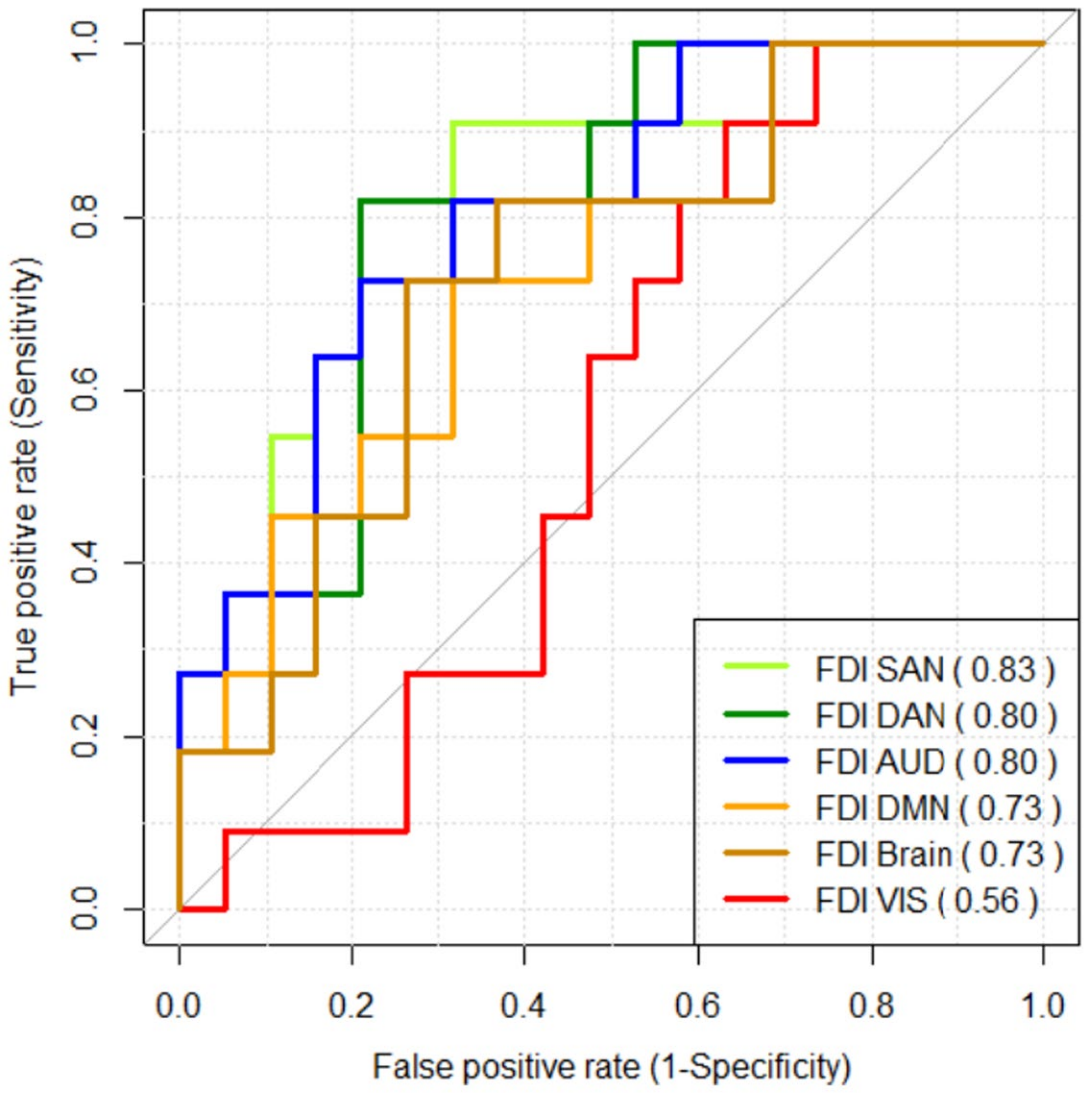
ROC curve analysis assessing the performance of FDI as a classifier for schizophrenia. The AUC value for each FDI measure is shown between brackets.

Finally, we assessed the performance of FDI as a potential feature for classification algorithms of schizophrenia using an ROC curve analysis. In this ROC analysis, we used directly to predict the corresponding FDI value, imposing the condition FDI SCZ ≤ FDI HC. Figure 7 shows the curves and AUC values obtained for the FDI measure in each resting-state network and the whole brain. High AUC values above 0.8 were obtained for FDI in networks SAN, DAN, and AUD.

## 4. Discussion and conclusion

Fractal dimension index is a measure of complexity which has been used until now for analyzing the brain activations extracted from very short epochs of electroencephalogram (EEG) signals induced by transcranial magnetic stimulation (Ruiz de Miras et al., 2019). In this study we hypothesized with the viability of the FDI methodology for analyzing both long periods of resting state EEG signals and localized regions of interest defined by functional networks. To this end, brain activations at each time sample were defined by a thresholding process (mean plus standard deviation) applied on 15,002 sources extracted from the EEG signal. FDI was then computed on the brain activations corresponding to each resting-state functional network by averaging all the FDI values obtained using a sliding window of 1 s on the epoch. This customized FDI methodology was successfully used for characterizing the brain dynamic changes in schizophrenia, a mental disorder associated with dysfunction in the information flow dynamics in the spontaneous brain networks. Our results demonstrated the ability of FDI to be applied on resting-state EEG signals from different regions of interest and revealed a localized decreasing complexity of neural activity of schizophrenia patients, mainly in the salience network.

We found that FDI was significantly lower in the SCZ group compared to HC for networks AUD, DAN, and SAN (see Figures 4, 5). However, the whole brain and the DMN and VIS networks did not statistically differ in FDI between the SCZ and HC groups. Decreased values of EEG complexity in schizophrenia patients were also reported in some recent studies (Goshvarpour and Goshvarpour, 2020; Kutepov et al., 2020; Aksentijevic et al., 2021). Results in Aksentijevic et al. (2021) showed that schizophrenia patients had lower values of change complexity than healthy subjects. Similar results were obtained by Kutepov et al. (2020) when comparing EEG complexity between schizophrenia patients and healthy controls by using approximated entropy, sample entropy, and multi-scale entropy. In Goshvarpour and Goshvarpour (2020), lower values of HFD were obtained in the patients group compared to normal subjects in all the 19 EEG channels recorded in resting-state. However, some other studies found higher values of complexity in the SCZ group compared to normal controls (Portnova and Atanov, 2018; Xiang et al., 2019). In Xiang et al. (2019), significantly higher values of fuzzy entropy in the frontal and occipital regions were obtained for schizophrenia patients in both conditions, baseline and auditory stimulus. Also, Portnova and Atanov (2018) found significantly higher values of HFD in the schizophrenia group for eight out of 19 EEG channels when the signal was recorded in resting-state. To these contradictory previous results our study adds a new standpoint by analyzing not the complexity of the EEG signal itself, but the 3D and 4D fractal dimensions of the spatiotemporal distribution of the brain activations extracted from the EEG.

According to Yang et al. (2015), from a systemic viewpoint the brain’s adaptability to an ever-changing environment is manifested in its complexity. From this point of view, reduced patterns in complexity could be markers of disease, or in terms of Costa et al. (2005) a generic feature of pathologic dynamics. A higher fractal dimension might indicate increased complexity, self-similarity, and possibly enhanced adaptability of neural networks. Conversely, a lower fractal dimension could imply a reduction in complexity, potentially related to disrupted neural communication changes (Iglesias-Parro et al., 2023). On the other hand, in a healthy brain, fractal dimension may reflect the brain’s ability to efficiently process information, respond to stimuli, and adapt to varying demands. A higher fractal dimension could indicate a balanced interplay between local and global neural processes, enabling cognitive flexibility, robustness, and efficient information processing (Ziukelis et al., 2022). Taken together, our results indicate that individuals diagnosed with schizophrenia compared to the control group presented, in most of the networks, a more rigid temporal resting-state dynamic. These results suggest a more rigid functional connectivity, with less adaptive capacity to segregate information and with a more stereotyped pattern, indicative of a network with less capacity to carry out functional adaptations in the connectivity of its nodes.

We also found strong negative correlations in the SCZ group between PANSS-P and the FDI of the whole brain and networks AUD, DAN, DMN, and SAN, and between PANSS-G and the FDI of networks AUD and SAN (see Figure 6 and Table 2). This result, which relates a decrease in FDI to an increase in the severity of the disease, is of great relevance because it indicates that FDI is a potential indicator in evaluating the clinical symptoms of schizophrenia. In clinical practice, schizophrenia is usually diagnosed by observing positive symptoms (delusions, hallucinations, speech disorders, and thought disorder) and negative symptoms (avolition, alogia, anhedonia, asociality, and blunted affect). However, positive and negative symptoms may have contrary effects on brain functional organizations (Zhou et al., 2007; Bernard et al., 2017), and it has been proposed that the possible reason for these inconsistent observations may be that positive and negative symptoms have separate mechanisms (Wang et al., 2023). Thus, our results are consistent with this differentiation, in that reduced network complexity was significantly correlated with the severity of positive psychotic symptoms but not with that of negative ones, inversely associated with cognitive measures. It would be possible that positive symptoms are physiologically characterized by neural networks with more rigid dynamics and a tendency to change in a more stereotypical way.

Since many recent studies have used EEG complexity measures as features in machine learning classifiers of schizophrenia (Racz et al., 2020; Barros et al., 2021; Tian et al., 2022; Ruiz de Miras et al., 2023a), we also assessed the performance of FDI as a potential feature for classifying schizophrenia patients by means of an ROC curve analysis (see Figure 7). Our results showed that FDI could be a promising feature to be used in classifiers based on EEG measures, especially when computed for the SAN network (AUC = 0.83). These results reinforce the hypothesis that cognitive networks—but not visual networks—would be differentially active in schizophrenia (Keane et al., 2022). Moreover, FDI as a feature could add a broader perspective when analyzing complexity in current machine learning classifiers which are based on linear and non-linear measures computed directly on the EEG signal, because the input EEG signal is used in FDI to extract the brain activations over time, which allows for analyzing 3D and 4D complexity through fractal dimension.

Fractal dimension index relies on repeatedly computing the fractal dimension of 3D and 4D point clouds, which is not trivial and could be computationally intensive. Nevertheless, we have already developed very efficient implementations of the computation of 3DFD and 4DFD through parallel execution on GPU. This software was already described and made publicly available in Ruiz de Miras et al. (2023b).

In conclusion, in this study we have presented a novel methodology, based on the FDI measure, for performing the complexity analysis of resting-state EEG signals. FDI performs a spatiotemporal analysis of brain dynamics by combining two components: 4DFD as a quantification of cortical integration, and HFD (3DFD) as a measure of cortical differentiation. Our complexity analysis based on the FDI of resting-state EEGs applied differentially on resting-state functional networks obtained very good results in differentiating schizophrenia patients from healthy subjects. Moreover, FDI was strongly correlated with clinical scores of schizophrenia. All these results suggest that FDI can be used for analyzing the complexity of brain dynamics from resting-state EEG signals, being a promising tool for obtaining a deeper understanding of the neurodegeneration present in neurological disorders such as schizophrenia.

### 4.1. Limitations

The impact of small sample sizes on study power is widely recognized, and it can lead to unreliable results, reducing the likelihood of detecting genuinely true effects. Considering this, we acknowledge that our results should be interpreted with caution due to the relatively small size of our sample. The primary reason for this limited sample size was the difficulty in accessing clinical samples, as many patients were hesitant to actively participate in experiments, particularly those involving EEG recordings. Moreover, our efforts to ensure consistency by conducting all EEG recordings on the same experimental set further constrained the sample size. While we faced challenges in encouraging control participants to visit the Hospital for the experiment, it was even more challenging to have patients from other hospitals come to our laboratory for data collection.

Undoubtedly, future research should focus on addressing these limitations to enhance the robustness and generalizability of findings. Whenever possible, supplementing public datasets with data from local research studies can enhance the robustness and generalizability of the results. Improving access to larger clinical samples and optimizing recruitment strategies for both control and patient groups will be crucial in overcoming these limitations and advancing our understanding of the topic. Future works should build upon these findings to refine methodologies and expand the participant pool for more comprehensive investigations.

## Data availability statement

The raw data supporting the conclusions of this article will be made available by the authors, without undue reservation.

## Ethics statement

The studies involving humans were approved by Universidad de Jaén (Spain)—Hospital San Agustín de Linares (Spain). The studies were conducted in accordance with the local legislation and institutional requirements. The participants provided their written informed consent to participate in this study.

## Author contributions

JR: conceptualization, methodology, software, formal analysis, investigation, data curation, writing original draft, and funding acquisition. AI-M and MS: methodology, investigation, resources, data curation, review, and editing original draft. SI-P: methodology, formal analysis, investigation, resources, data curation, writing original draft, and funding acquisition. All authors contributed to the article and approved the submitted version.

## Funding

This work is part of the research project PID2019-105145RB-I00 supported by the Spanish Government (MCIN/AEI/10.13039/501100011033).

## Conflict of interest

The authors declare that the research was conducted in the absence of any commercial or financial relationships that could be construed as a potential conflict of interest.

## Publisher’s note

All claims expressed in this article are solely those of the authors and do not necessarily represent those of their affiliated organizations, or those of the publisher, the editors and the reviewers. Any product that may be evaluated in this article, or claim that may be made by its manufacturer, is not guaranteed or endorsed by the publisher.
